# Efficacy of praziquantel treatment regimens in pre-school and school aged children infected with schistosomiasis in sub-Saharan Africa: a systematic review

**DOI:** 10.1186/s40249-018-0448-x

**Published:** 2018-07-05

**Authors:** Muhubiri Kabuyaya, Moses John Chimbari, Samson Mukaratirwa

**Affiliations:** 1 Discipline of Public Health Medicine, Howard College, University of KwaZulu-Natal, P.O Box 4041, Durban, South Africa; 2 College of Health Sciences, University of KwaZulu-Natal, Durban, South Africa; 3 School of Life Sciences, University of KwaZulu-Natal, Durban, South Africa

**Keywords:** Praziquantel, Efficacy, Resistance, *Schistosoma mansoni*, *Schistosoma haematobium*, Sub-Saharan Africa

## Abstract

**Background:**

Schistosomiasis is a serious public health burden in sub-Saharan Africa. Praziquantel is the only drug recommended by the World Health Organization to treat both urogenital and intestinal schistosomiasis. The reliance on a single drug to treat a disease with such a huge burden has raised concerns of possible drug resistance mainly in endemic areas. This systematic review was conducted to identify gaps and recent progress on the efficacy of different regimens of praziquantel in treating schistosomiasis among children in sub-Saharan Africa where *Schistosoma mansoni* and *S. haematobium* are endemic.

**Main text:**

A literature search of peer-reviewed journals was done on Google Scholar, MEDLINE (under EBSCOhost) and PubMed databases using pre-defined search terms and Boolean operators. The search included studies published from 2008 to 2017 (August) with emphasis on the efficacy of praziquantel on *S. haematobium* and *S. mansoni* infections among preschool and school children. Nineteen publications satisfied the inclusion criteria for the review. The studies reviewed were from 10 sub-Saharan African countries and 7/19 of the studies (37%) were conducted in Uganda. Seven studies (37%) focused on *Schistosoma mansoni*, 6/19 (31.5%) on *S. haematobium* and another 6 on mixed infection. A single standard dose of 40 mg/kg body weight was the most used regimen (9) followed by the repeated single standard dose assessed for efficacy at 3–4 weeks post-treatment.

**Conclusions:**

A repeated standard dose of 40 mg/kg achieved satisfactory efficacy compared to a single dose against both parasite species. However, findings on efficacy of repeated doses in co-infection of *S. mansoni* and *S. haematobium* were not conclusive. Praziquantel administrated at 60 mg/kg was slightly more efficacious than the 40 mg/kg standard dose. Minor and transitory side-effects were reported for both regimens. The review indicates that further investigations are necessary to conclusively determine efficacy of praziquantel on coinfection of *S. haematobium* and *S. mansoni* to formulate concrete guidelines on the use of repeated doses at 40 or 60 mg/kg for treating schistosomiasis. We recommend the use of the egg reduction rate (ERR) formula recommended by the WHO for assessing praziquantel efficacy in order for the results to be comparable for different regions.

**Electronic supplementary material:**

The online version of this article (10.1186/s40249-018-0448-x) contains supplementary material, which is available to authorized users.

## Multilingual abstracts

Please see Additional file 1 for translations of the abstract into six official working languages of the United Nations.

## Background

*Schistosoma* infection is a worldwide public health problem, particularly in sub-Saharan Africa where approximately 90% of the infections are found [1]. Both *Schistosoma haematobium* and *Schistosoma mansoni* are responsible for the burden of the disease although *S. haematobium* is more prevalent in the sub-Saharan region. In 2012, at least 249 million people required preventive treatment and only 42.1 million were reported to have been treated [2]. To date, praziquantel administered at the standard single oral dose of 40 mg/kg body weight is the mainstay drug recommended by WHO for chemo-preventive therapy [2, 3]. This dosage has been largely used in mass drug administration (MDA) programmes in endemic countries to reduce the morbidity of the disease [4].

In sub-Saharan Africa, studies have shown that praziquantel drastically reduces morbidity and transmission of schistosomiasis. It has a high cure rate (CR) and satisfactory egg reduction rate (ERR) [5, 6]. However, failure/resistant cases have been reported after the use of a single standard dose of praziquantel at 40 mg/kg body weight. A repeated standard dose regimen has been shown to be more efficacious than the single dose [7]. Even when the repeated dose was used, few treated cases continued to release viable eggs. This prompted trials with a single dose of 60 mg/kg body weight to prevent failure/resistant cases. Researchers that conducted comparative studies of praziquantel with the standard dose of 40 mg/kg versus 60 mg/kg at a split dose [8, 9] reported divergent results. Belizario et *al.* reported similar efficacy on *S. mansoni* with both dose therapies [8] and significantly higher but mild and transient side effects with 60 mg/kg regimen [8]. In contrast, Coulibaly et al*.* showed that 60 mg/kg was more efficacious than 40 mg/kg [9].

Various factors such as species of parasite [7], parasite stage [10] and infection intensity have been attributed to influence the treatment outcomes with praziquantel with regard to the CR and ERR. Praziquantel is reported to be more efficacious on *S. japonicum* than it is on *S. mansoni*. *S. haematobium* has the lowest CR among mixed infections of *S. haematobium* and S*. mansoni* [7, 11]. It is also known that praziquantel is not effective on immature worms [12]. Moreover, schistosome resistance to praziquantel treatment following its repeated dose use has been reported in field studies [10].

Since there is no vaccine to prevent *Schistosoma* infection, regular monitoring of the efficacy of praziquantel is crucial. Studies on *S. mansoni* and *S. japonicum* focusing on the genetic diversity of the parasite as the determinant praziquantel efficacy have been conducted in Japan, China and the Philippines. Since praziquantel is not active on immature worms [12], a combination of praziquantel with antimalarial drugs (artemether, artesunate) which kill immature worms has been suggested as a comprehensive cure [12, 13]. Phase 3 schistosome vaccine clinical trials are in progress [14]. In sub-Saharan Africa, where *S. mansoni* and *S. haematobium* are endemic, particularly among children, studies on the efficacy of praziquantel are limited and the few studies that investigated the relationship between morbidity due to *S. haematobium* and the genetic variation have reported conflicting results [15, 16]. This highlights the need for further investigations on the efficacy/failure of different doses of praziquantel against *S. haematobium* and *S. mansoni* in endemic areas of sub-Saharan African. In this paper we reviewed the status of use of praziquantel and identified gaps in the treatment of schistosomiasis in the sub-Saharan region? Electronic searches focusing on the efficacy of different regimens of praziquantel in treating schistosomiasis among children in sub-Saharan Africa were done to extract literature on the subject.

## Main text

### Review methods

#### Search strategy

A systematic electronic search of literature on PubMed, MEDLINE and Google Scholar databases was carried out using following terms and Boolean operators: praziquantel AND efficacy AND resistance AND schistosomiasis AND *Schistosoma haematobium* OR *Schistosoma mansoni* AND sub-Saharan Africa. At the initial stage of identifying articles, titles and abstracts were used to screen relevant papers. Relevant abstracts were further assessed for inclusion in the list of full text articles. The full texts were further assessed for eligibility in the review. All records were managed in Endnote version X7 (Clarivate Analytics, Philadelphia, PA, USA). The review included research articles published within a 10 year period from 2008 to 2017 (August).

#### Inclusion and exclusion criteria

Publications in peer-reviewed journals that focused on field studies on the efficacy and resistance/failure of praziquantel on *S. haematobium and S. mansoni* treatment among children in sub-Saharan Africa were included in the review. Studies that reported on interventional studies conducted among children less than 18 years old were also included in the review. Studies in which praziquantel was administrated at 40 mg single standard dose and/or repeated standard dose or at escalating dose (20/40/60 mg/kg) were added to the list of eligible articles.

The following studies were excluded:

- Reports on work that involving both children and adults;
- Studies that reported on the efficacy of praziquantel combined with other anti-schistosomal drugs;
- Studies that assessed the efficacy of praziquantel in the co-infection settings of schistosomiasis with other parasitic diseases like soil transmitted helminthic diseases;
- Laboratory experimental studies on praziquantel efficacy;
- Studies in which praziquantel efficacy was assessed after more than nine weeks post first administration.

Quality assessment of the studies was performed using the Study Quality Assessment Tool for Quality Assessment of Controlled Intervention Studies. Individual studies were assigned a score that was computed using different parameters in line with the review objectives. The responses were scored 0 for “No” and 1 for “Yes”. Total scores ranged between 0 and 10; 1–4 = (Low); 5–7 = (Moderate) and 8–10 = (High). The overall score was moderate.

## Results

**Fig. 1:**
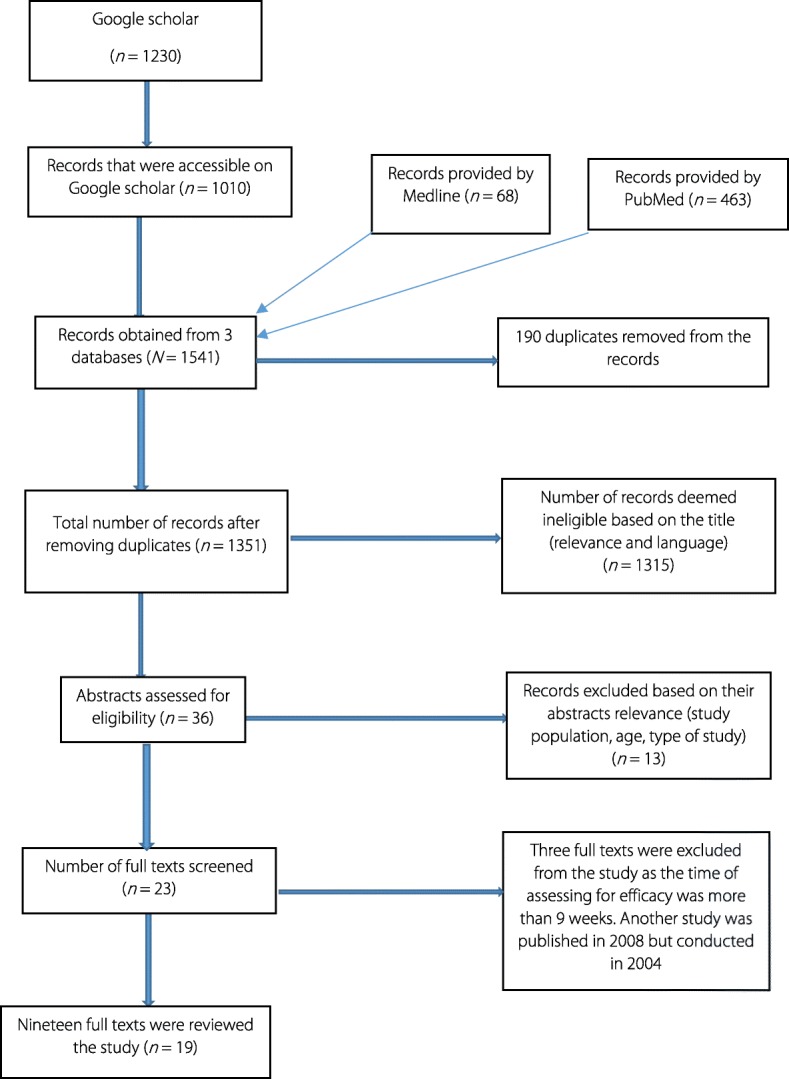
PRISMA diagram showing the process of studies search. PRISMA: Preferred Reporting Items for Systematic Reviews and Meta-Analyses

The literature search from Google Scholar provided one thousand two hundred and thirty (1230) records of which only 1010 were accessible. The search setting for ten results per page could not allow to go further than the 100^th^ webpage. We obtained 463 records from the PubMed database and sixty eight (68) from MEDLINE. Overall, the electronic search provided a total of *n* = 1541 hits (Fig. 1). Hundred and ninety duplicate articles were removed from the records. During the screening of titles 1315 were deemed ineligible based on their relevance and the language in which they were published. Therefore, they were excluded from the screening process for abstracts. Out of the remaining 36 abstracts that were deemed relevant, 13 were excluded as they did not focus specifically on the efficacy of praziquantel against *S. haematobium* and/or *S. mansoni* in sub-Saharan Africa in a field setting. This gave a total of 23 studies eligible for full text screening. The review included interventional studies (cohort and randomised control). Excluded studies were those that reported either on the efficacy of praziquantel in combination with other medications or experimental laboratory studies or reviews. Studies in which the study population comprised children and adults were also excluded. Out of the twenty three studies whose full texts were reviewed, three were removed as the time line of assessing for praziquantel efficacy did not meet the inclusion criteria (3–9 weeks). An additional study was removed as it was published in 2008 but was carried out in 2004. Therefore, nineteen (19) publications were deemed relevant for final inclusion and were reviewed (Fig. 1).

### Efficacy of praziquantel by age group

The reviewed studies were conducted among children. School-going children were the most represented (eight studies) followed by pre-school children (seven studies). Four studies involved both age groups. The highest number of the studies (nine) were conducted between 2012 and 2013.

The cure rate had been largely used in assessing praziquantel efficacy in the past. The use of the egg reduction rate was recently recommended [17]. In most studies that were carried out among school going children, the efficacy of praziquantel improved significantly after the repeated standard dose of 40 mg/kg body weight [18–23]. Higher cure rates were observed among people infected with *S. mansoni* those infected with *S. haematobium* infection [24]. Pre-school children were included among the target group for schistosmiasis treatment after realizing that they were a high risk group as well. [25]. Praziquantel administered at 40 mg/kg single dose demonstrated satisfactory efficacy among children [26–28]. Efficacy was lower in children that received multiple rounds of praziquantel treatment than in those that did not receive any treatment before [29]. In response to the pressing need of standardizing treatment in pre-school children, alternative paediatric forms of praziquantel have been on trial. Crushed praziquantel has been reported to have satisfactory efficacy for all species [30] while praziquantel syrup (Epiquantel®) showed diminished efficacy [31] for reasons not known.

### Distribution of studies by country and species

Of the 19 studies reviewed, the majority of them (7/19 = 37%) were carried out in Uganda where *S. mansoni* was the predominant species (Additional file 2: Table S1). Another study on *S. mansoni* infection carried out in Ethiopia [19] indicated an East Africa dominance in distribution of *S. mansoni.* Elsewhere in other countreis of sub-Saharan Africa *S. haematobium* and mixed infections were found (Additional file 3: Table S2 and Additional file 4: Table S3). As shown in Additional file 4: Table S3, of the 6 mixed infections that were reported, 2 were from Niger [31, 32] and one from Cameroun, Ivory Coast, Senegal and Zimbabwe [23, 24, 28, 30]. The rest of the infections were due to *S. haematobium* (Additional file 3: Table S2).

### Efficacy of praziquantel on *S. haematobium* and *S. mansoni*

*S. haematobium* and *S. mansoni* both exist separately or in co-infection in sub-Saharan countries. Praziquantel is efficacious on all species, therefore, it is the only recommended drug for preventing and treating schistosomiasis [2] despite some observed low cure rates and possible resistance recently reported. This review shows that for most of the studies praziquantel had high efficacy on both species [19, 22, 27, 28, 29, 34]. However, higher efficacy was reported against *S. haematobium* [30–32] than it was with *S. mansoni* [24] in co-infection settings as shown in Additional file 4: Table S3.

### Efficacy of different dose regimens of praziquantel

Praziquantel administered at 40 mg/kg body weight is the standard dose recommended by WHO for chemotherapy in treating schistosomiasis. Articles reviewed mainly reported on the efficacy of different dosages of praziquantel used against both *S. haeamtobium* and/or *S. mansoni* in sub-Saharan Africa. Most studies used single dose (9/19, 47%) followed by studies that used single dose versus repeated standard dose (6/19, 32%). Repeated standard dose was administered in three studies while one study used the escalating dose (20/40/60 mg/kg). The efficacy of praziquantel varied significantly depending on whether it was single or repeated dose. Overall, the repeated dose was more efficacious compared to the single dose. Cure rates and egg reduction rates were low or moderate after the single dose and improved after the administration of the second dose [23, 27, 31–33]. In some studies, praziquantel given at single dose revealed high cure rates and egg reduction rates [19, 28, 34, 35] while they were low in others [31, 36]. Escalating dose of praziquantel was more efficacious at high dose than at low dose [9].

## Discussion

The study reviewed the efficacy of different doses of praziquantel against *S. mansoni* and *S. haematobium* infection in endemic settings in sub-Saharan Africa. Our discussion is based on the used dose, the targeted species and the time of assessment post-treatment. An emphasis was also given to studies conducted in high endemic zones where several rounds of mass drug administration were done.

Praziquantel administered at a standard dose of 40 mg/kg was shown to be effective against *Schistosoma* infection. However, a low cure rate that may require a second or high dose to clear the infection was reported [36]. This may be because praziquantel kills mature schistosomes while immature worms are not affected but later become mature and release eggs at a later stage. Infection may also persist after praziquantel treatment due to the reduced susceptibility of juvenile parasites to the drug [37]. Such persistent infections may then contain a significant amount of juvenile schistosomes that are refractory to praziquantel at the standard dose leading to lower cure rate and egg reduction rate. In high endemic areas where school-going children had been subjected to several rounds of mass drug administration (MDA), it was evident that praziquantel at the standard dose was less efficacious among those that received higher rounds of praziquantel (8 to 9) compared to those that received lesser rounds of treatment (1 and 5) [38]. Thus, the use of praziquantel in MDA campaigns has raised concerns on the possibility of development of drug resistance. It is however important to realize that the parasite may acquire tolerance to praziquantel that needs to be distinguished from resistance. Repeated doses of praziquantel have been reported to improve the outcomes of infection, post treatment [7]. Follow-ups after large scale intervention such as MDA are needed since praziquantel is the only recommended drug for schistosomiasis at 40 mg/kg body weight and in most cases does not completely clear the parasite.

Studies that used repeated standard dose of praziquantel [20, 33] showed satisfactory efficacy after the second dose. These findings corroborate with those found in another study [7]. Administration of the second dose was intended to target immature worms missed by first treatment and that was done 3–4 weeks following the initial treatment. Moreover, the outcomes of praziquantel efficacy varied depending on whether it was a single infection or mixed infection. Studies that focused on one species reported satisfactory efficacy [20, 33, 39] while those focused on co-infection (*S. haematobium* and *S. mansoni)* had inconsistent results [24, 32]. The period of assessment between the first dose and follow up might have not been sufficient to allow complete release of dead eggs from the human host. However, the underlying cause of this reduced efficacy among mixed infection needs to be elucidated. Despite the use of the second dose, few refractory cases were still observed. *Schistosome* infections may persist after the treatment due to the reduced susceptibility of immature parasites to the drug [37] and may develop tolerance or resistance over time. This may be the same mechanism that schistosomes acquire tolerance/resistance in endemic areas after several rounds of MDAs.

In a study that used an escalating dose, the outcomes of praziquantel efficacy gradually improved with increasing dosage [9]. The initial dose of 20 mg/kg body weight was lower than the recommended dose of 40 mg/kg to kill schistosomes and showed low efficacy. Consistent with the above, results from a laboratory experiment showed that the use of praziquantel, especially at a lower dose than the recommended curative dose may lead to development of resistance to the drug in future generations [40]. Another study that compared praziquantel given at 40 mg/kg and 60 mg/kg single doses reported satisfactory efficacy with both regimens. However, there was no significant difference between the doses but more minor and transient side effects were observed at 60 mg/kg regimen [8].

This review showed that praziquantel was efficacious on both *S. haematobium* and *S. mansoni* with the former being more sensitive to praziquantel. In another comparative study, the efficacy of praziquantel was shown to be higher against *S. japonicum* than other species [11]. Factors responsible for this difference are not clear and need further investigation in co-infection settings.

## Conclusions

This review revealed that praziquantel administrated at a repeated standard dose of 40 mg/kg was more efficacious than the standard single dose of 40 mg/kg. The outcomes of praziquantel efficacy depended on whether it was mixed infection of *S. haematobium* and *S. mansoni* or a single focus of infection. The efficacy of praziquantel in mixed infection foci required at least two rounds of standard dose at three weeks interval. Praziquantel given at 60 mg/kg single dose was shown to be slightly more efficacious than other regimens. *S. haematobium* was shown to be more sensitive to praziquantel than *S. mansoni*. Praziquantel showed moderate efficacy in areas that had received multiple MDA. The review suggests that follow ups should be done in endemic area after mass drug administration as recently initiated in Zimbabwe by Mutapi and Mduluza (personal communication). The use of repeated doses of 40 mg/kg and the high dose (60 mg/kg) need further investigations before being recommended for use in control programmes in endemic areas of sub-Saharan Africa to prevent possible resistance to praziquantel.

## Additional files

Additional file 1: Multilingual abstracts in the six official working languages of the United Nations. (PDF 417 kb)

Additional file 2: **Table S1**. Summary of findings on the use of praziquantel against *Schistosoma mansoni* between 2008–2017 in sub-Saharan Africa. (DOCX 25 kb)

Additional file 3: **Table S2**. Summary of findings on the use of praziquantel against *Schistosoma haematobium* between 2008–2017 in sub-Saharan Africa. (DOCX 22 kb)

Additional file 4: **Table S3**. Summary of review of findings on the use of praziquantel on co-infection with *Schistosoma haematobium* and *S. mansoni* between 2008–2017 in sub-Saharan Africa. (DOCX 22 kb)

## Abbreviations

CR: Cure rate
ERR: Egg reduction rate
GM: Geometric mean
MDA: Mass drug administration
PZQ: Praziquantel
WHO: World Health Organisation
