# Correction: Efficacy of praziquantel treatment regimens in pre-school and school aged children infected with schistosomes in sub-Saharan Africa: a systematic review

**DOI:** 10.1186/s40249-023-01064-5

**Published:** 2023-02-23

**Authors:** Muhubiri Kabuyaya, Moses John Chimbari, Samson Mukaratirwa

**Affiliations:** 1 Discipline of Public Health Medicine, Howard College, University of KwaZulu-Natal, P.O Box 4041, Durban, South Africa; 2 College of Health Sciences, University of KwaZulu-Natal, Durban, South Africa; 3 School of Life Sciences, University of KwaZulu-Natal, Durban, South Africa

**Correction: Infectious Diseases of Poverty (2018) 7:73** **https://doi.org/10.1186/s40249-018-0448-x**

Following publication of the original article [1], the Editorial Office reported that the word “schistosomiasis” in the title should be changed to “schistosomes” in order to appropriately summarize the content of the research.

The correct title should read: Efficacy of praziquantel treatment regimens in pre-school and school aged children infected with schistosomes in sub-Saharan Africa: a systematic review.

The original article [1] has been updated.
