# Supplementary material for: Efficacy of praziquantel treatment regimens in pre-school and school aged children infected with schistosomiasis in sub-Saharan Africa: a systematic review
Source: Infect Dis Poverty. 2018 Jul 5;7:73. doi: 10.1186/s40249-018-0448-x (PMC6036702; doi:10.1186/s40249-018-0448-x)
Supplement: Supplementary file 1 — Multilingual abstracts in the six official working languages of the United Nations. (PDF 417 kb) [file 40249_2018_448_MOESM1_ESM.pdf]

Translation of the abstract into the five official working languages of the United Nations

فعالية نظم علاج البرازيكوانتيل لدى الأطفال في سن ما قبل المدرسة والمدرسة المصابون بداء البلهارسيا في جنوب الصحراء الكبرى بأفريقيا: مراجعة منهجية

موهبيري كابويايا، موسى جون شيمباري وسامسون موكاراتيروا

الملخص
الخلفية: داء البلهارسيا عبء خطير على الصحة العامة في جنوب الصحراء الكبرى بأفريقيا. البرازيكوانتيل هو الدواء الوحيد الذي أوصت به منظمة الصحة العالمية لعلاج كل من البلهارسيا البولية والمعوية. وقد أثار الاعتماد على دواء واحد لعلاج مرض يحمل هذا العبء الثقيل المخاوف من مقاومة الدواء المحتملة أساساً في المناطق الموبوءة . أجريت هذه المراجعة المنهجية لتحديد الثغرات والتقدم الذي حدث مؤخراً في فعالية الأنظمة المختلفة من البرازيكوانتيل في علاج داء البلهارسيا بين الأطفال في جنوب الصحراء الكبرى بأفريقيا حيث تستوطن البلهارسيا المنسونية والبلهارسيا الدموية .
المتن: تم إجراء بحث في المطبوعات من المجلات التي تمت مراجعتها من قبل الزملاء على Google Scholar و Medline (تحت EBSCOhost) وقواعد بيانات PubMed باستخدام مصطلحات بحث محددة مسبقًا ومشغلات Boolean. شمل البحث دراسات نشرت من 2008 إلى 2017 (أغسطس) مع التركيز على فعالية البرازيكوانتيل على إصابات البلهارسيا الدموية والبلهارسيا المنسونية بين الأطفال في مرحلة ما قبل المدرسة وأطفال المدارس. استوفت 19 مطبوعة معايير الاشتمال للمراجعة. كانت الدراسات التي تمت مراجعتها من 10 بلدان أفريقية بجنوب الصحراء الكبرى و19/7 من الدراسات (37 ٪) أجريت في أوغندا. ركزت سبع دراسات (37٪) على البلهارسيا المنسونية ، 19/6 (31.5٪) على البلهارسيا الدموية و 6 أخرى على العدوى المختلطة. كانت الجرعة القياسية الواحدة 40 ملغ/كغ من وزن الجسم هي النظام الأكثر استخدامًا (9) متبوعة بالجرعة المعيارية المتكررة التي تم تقييمها لتحقيق الفعالية عند 3-4 أسابيع بعد المعالجة.
الاستنتاجات: حققت الجرعة القياسية المتكررة من 40 ملغ/ كغ فعالية مرضية مقارنة بالجرعة الواحدة ضد كلا نوعي الطفيلي. ومع ذلك ، فإن النتائج حول فعالية الجرعات المتكررة في العدوى المشتركة للبلهارسيا المنسونية والبلهارسيا الدموية لم تكن حاسمة. كان استخدام البرازيكوانتيل في 60 ملغم / كغم أكثر فعالية قليلاً من الجرعة القياسية 40 ملغ / كغ. تم الإبلاغ عن آثار جانبية صغيرة وعابرة لكل من النظامين. تشير المراجعة إلى ضرورة إجراء مزيد من التحقيقات لتحديد فعالية البرازيكوانتيل على الإصابة المشتركة بالبلهارسيا الدموية والبلهارسيا المنسونية لصياغة إرشادات محددة بشأن استخدام الجرعات المتكررة عند 40 أو 60 مغ / كغ لعلاج داء البلهارسيا. نوصي باستخدام صيغة معدل تخفيض البيض (ERR) التي أوصت بها منظمة الصحة العالمية لتقييم فعالية البرازيكوانتيل من أجل أن تكون النتائج قابلة للمقارنة في المناطق المختلفة.

Translated from English version into Arabic by Free Bird and Faheem Ahmad, through

TRANSLATORS
WITHOUT BORDERS

吡喹酮治疗方案对撒哈拉以南非洲地区学前/龄血吸虫病患儿的疗效：一项系统综述

Muhubiri Kabuyaya, Moses John Chimbari and Samson Mukaratirwa

摘要：
引言：在撒哈拉以南的非洲地区，血吸虫病是造成了严重的公共卫生负担。吡喹酮是世界卫生组织推荐用于治疗泌尿生殖道和肠道血吸虫病的唯一药物。人们已经开始关注依靠单一药

物治疗具有如此重大负担的疾病是否会在主要流行区产生耐药性。本文系统性回顾了在撒哈拉以南非洲的曼氏血吸虫和埃及血吸虫流行区中吡喹酮对儿童血吸虫病的疗效差异和最新进展。
正文：我们在 Google Scholar、MEDLINE（隶属 EBSCOhost）和 PubMed 数据库中使用预定义的搜索术语和 Boolean 运算符对同行评审期刊进行文献检索。我们检索出 2008 年至 2017 年（8 月）发表的关于使用吡喹酮治疗感染埃及和曼氏血吸虫的学前和学龄患儿的效果研究相关文章。本综述共纳入 19 份研究，其中 10 份来自非洲撒哈拉以南地区国家，7 项研究（37%）在乌干达完成。7/19 研究（37%）涉及曼氏血吸虫，6/19（31.5%）涉及埃及血吸虫，其余 6 份为混合感染。最常用的治疗方案按照吡喹酮单次标准剂量 40mg/kg（9）两次服药， 3-4 周评估其疗效。
结论：吡喹酮服用两次比单次服药对两种血吸虫疗效均更好。然而，两次服药对曼氏和埃及血吸虫合并感染的疗效并不明确。以 60 mg/kg 比 40 mg/kg 标准剂量给药稍微更有效。两种治疗方案均报道存在轻度和短暂的副作用。本综述表明，需要进一步的研究来明确吡喹酮对埃及和曼氏血吸虫合并感染的疗效，以制定关于两次服用 40mg/kg 或 60 mg/kg 吡喹酮治疗血吸虫病的具体指南。我们建议使用世界卫生组织推荐的减卵率（ERR）公式来评估吡喹酮疗效，以使不同地区的结果具有可比性。

Translated from English version into Chinese by Peng Song, edited by Jin Chen

CHINESE CENTER FOR DISEASE CONTROL AND PREVENTION
NATIONAL INSTITUTE OF PARASITIC DISEASES

Étude de l'efficacité des différentes posologies du traitement praziquantel chez les enfants d'âge préscolaire et scolaire infectés par la schistosomiase en Afrique subsaharienne

Muhubiri Kabuyaya, Moses John Chimbari et Samson Mukaratirwa

Résumé

Contexte : La schistosomiase est un problème de santé publique majeur en Afrique subsaharienne. Le praziquantel est le seul médicament recommandé par l'Organisation Mondiale de la Santé pour traiter à la fois la schistosomiase urogénitale et intestinale. Mais la dépendance à l'égard d'un seul médicament pour traiter cette maladie aux effets dévastateurs soulève l'inquiétude de certains face à une éventuelle résistance au médicament, en particulier dans les régions endémiques. Cette étude a été menée pour identifier les lacunes et les progrès récents sur l'efficacité des différentes posologies du praziquantel dans le traitement de la schistosomiase chez les enfants en Afrique subsaharienne, où le *Schistosoma mansoni* et le *Schistosoma haematobium* sont endémiques.
Corps du texte principal : Une recherche dans des journaux approuvés par des comités de lecture scientifiques a été faite sur Google Scholar, Medline (avec EBSCOhost) et sur la base de données PubMed en utilisant des termes de recherche prédéfinis et des opérateurs booléens. La recherche s'est focalisée sur des études publiées entre 2008 et le mois d'août 2017 mettant l'accent sur l'efficacité du praziquantel sur les infections causées par le *S. haematobium* et le *S. mansoni* parmi les enfants d'âge préscolaire et scolaire. Au final, dix-neuf publications ont satisfait aux critères d'inclusion de la recherche. Les études examinées provenaient de 10 pays d'Afrique subsaharienne et 37 % d'entre elles (soit 7/19) ont été menées en Ouganda. Sept études (soit 37 %) portent sur le*S. mansoni*, 6 (soit 31.5 %) sur le *S. haematobium* et 6 autres concernent des infections diverses. La

posologie la plus utilisée (dans 9 des études) était la dose standard unique de 40 mg/kg de poids corporel, suivie du renouvellement de la dose standard unique dont l'efficacité a été évaluée 3 à 4 semaines après le traitement.
Conclusions de l'étude : Une dose standard renouvelée de 40 mg/kg permet d'obtenir des résultats satisfaisants comparée à une dose unique, et ce contre les deux espèces de parasite. Toutefois, les résultats sur l'efficacité des doses répétées contre l'infection simultanée du *S. mansoni* et du *S. haematobium* n'ont pas été concluantes. Aussi, le praziquantel administré à 60 mg/kg s'est avéré légèrement plus efficace que la dose standard de 40 mg/kg. Il faut également noter que des effets secondaires mineurs et passagers ont été signalés pour les deux posologies. Cette étude montre que d'autres recherches sont nécessaires pour déterminer de façon concluante l'efficacité du praziquantel sur l'infection simultanée du *S. haematobium* et du *S. mansoni* et ce afin de formuler des directives concrètes sur l'utilisation de doses répétées à 40 ou à 60 mg/kg pour le traitement de la schistosomiase. Nous recommandons ainsi l'utilisation du calcul du taux de réduction des oeufs de parasite de l'OMS pour évaluer l'efficacité du praziquantel afin que les résultats puissent être comparables dans différentes régions du monde.

Translated from English version into French by Karine H and Camille Perrolle, through

TRANSLATORS
WITHOUT BORDERS

Систематический обзор эффективности схем лечения празиквантелом детей дошкольного и школьного возраста, инфицированных шистосомозом, в странах Африки, расположенных к югу от Сахары

Мухубири Кабуяя, Мозес Джон Чимбари и Самсон Мукаратирва

Аннотация

Справочная информация:Шистосомоз является серьезной проблемой для здоровья населения в странах Африки, расположенных к югу от Сахары. Празиквантел — это единственное лекарственное средство, рекомендованное Всемирной организацией здравоохранения для лечения мочеполового и кишечного шистосомоза. Зависимость от одного лекарственного средства для лечения болезни, которая представляет такую серьезную проблему, вызывает обеспокоенность по поводу возможной резистентности к лекарственному средству, главным образом в эндемических областях. Настоящий систематический обзор был выполнен с целью выявления пробелов и достигнутого в последнее время прогресса в эффективности различных схем лечения шистосомоза у детей, проживающих в странах Африки, расположенных к югу от Сахары, где *широко распространены* шистосома Мансони *и* шистосома гематобиум.
Основная часть: Мы провели информационно-поисковую работу по рецензируемым научным журналам в базах данных Google Scholar, Medline (под EBSCOhost) и PubMed с использованием заданных критериев поиска и логических операторов. В своей

информационно-поисковой работе мы использовали исследования, опубликованные в период с 2008 по 2017 год (август). Особое внимание было уделено эффективности празиквантела в отношении инфекций *шистосома гематобиум* и *шистосома Мансони* среди детей дошкольного и школьного возраста. Девятнадцать публикаций удовлетворили наши критерии включения с целью произведения оценки. Проанализированные исследования проводились в 10 странах Африки, расположенных к югу от Сахары, в то время как 7 из всех исследований (37%) — в Уганде. Семь исследований (37%) были посвящены *шистосоме Мансони*, 6 из 19 (31.5%) — *шистосоме гематобиум* , а остальные 6 — смешанной инфекции. Единоразовая стандартная доза 40 мг на кг массы тела являлась самой широко используемой схемой лечения (9), за ней следовала схема лечения, в которой проводилось повторное администрирование единоразовой стандартной дозы с последующей оценкой на эффективность через 3-4 недели после первичного администрирования.

Заключение: Повторная стандартная доза 40мг на кг массы тела давала больше положительных результатов в сравнении с единоразовой стандартной дозой в отношении паразитов обоих видов. Однако данные об эффективности повторных доз при коинфецировании *шистосомой Мансони* и *шистосомой гематобиум* не являются исчерпывающими. Администрирование доз празиквантела по 60 мг/кг были немного более эффективны, чем стандартных доз по 40 мг/кг. При обоих методах лечения сообщалось о возникновении незначительных и ограниченных по времени побочных эффектов. Обзор показывает, что необходимы дальнейшие исследования для окончательного определения эффективности празиквантела при коинфецировании *шистосомой гематобиум* и *шистосомой Мансони* с целью предоставления конкретных рекомендаций в отношении использования повторных доз по 40 мг/кг или 60 мг/кг при лечении шистосомоза. Мы рекомендуем использовать формулу показателя снижения количества яиц (ПСКЯ), рекомендованную ВОЗ для оценки эффективности празиквантела, чтобы обеспечить сопоставимость результатов по различным регионам.

Translated from English version into Russian by Ekaterina Grebenshchikova and Liudmila Tomanek, through

TRANSLATORS
WITHOUT BORDERS

La eficacia los regímenes de tratamiento con praziquantel en niños en edad de educación preescolar y primaria infectados con esquistosomiasis en África subsahariana: una revisión sistemática

Muhubiri Kabuyaya, Moses John Chimbari y Samson Mukaratirwa

Resumen

Antecedentes: la esquistosomiasis es una carga grave para la salud pública en África subsahariana. El praziquantel es la única droga recomendada por la Organización Mundial de la Salud para tratar

tanto la esquistosomiasis urogenital como intestinal. La dependencia de una sola droga para tratar una enfermedad con semejante carga ha generado inquietudes sobre una posible resistencia a la droga, en especial en las áreas endémicas. Esta revisión sistemática se realizó para identificar las brechas y el progreso reciente en la eficacia de los diferentes regímenes de praziquantel para tratar la esquistosomiasis en los niños de África subsahariana donde *la Schistosoma mansoni* y *la S. haematobium* son endémicas.

Texto principal: se realizó una búsqueda bibliográfica de revistas arbitradas en Google Scholar, Medline (por EBSCOhost) y las bases de datos de PubMed a través del uso de términos de búsqueda ya definidos y operadores booleanos. La búsqueda incluyó estudios publicados desde el 2008 hasta agosto del 2017 con un enfoque en la eficacia del praziquantel en las infecciones de *S. haematobium* y *S. mansoni* en los niños en edad de educación preescolar y primaria. Diecinueve publicaciones cumplieron con el criterio de inclusión para este estudio. Los estudios revisados fueron de 10 países de África subsahariana y 7/19 de los estudios (37 %) se llevaron a cabo en Uganda. Siete estudios (37 %) se enfocaron en la *Schistosoma mansoni*, 6/19 (31,5 %) se enfocaron en la *S. haematobium* y otros seis, en infecciones mixtas. Una dosis estándar única de 40 mg/kg de peso corporal fue el régimen más utilizado (9), seguido por la dosis estándar única evaluada por eficacia a las 3-4 semanas luego del tratamiento.

Conclusiones: una dosis estándar repetida de 40 mg/kg logró una eficacia satisfactoria comparada con una dosis única contra ambas especies de parásitos. Sin embargo, los resultados sobre la eficacia de las dosis repetidas en las infecciones conjuntas de *S. mansoni* y *S. haematobium* no fueron concluyentes. El praziquantel administrado en 60 mg/kg fue apenas más eficaz que la dosis estándar de 40 mg/kg. Se presentaron efectos secundarios menores y transitorios en ambos regímenes. El estudio indica que se necesitan más investigaciones para determinar, de forma concluyente, la eficacia del praziquantel en las infecciones conjuntas de *S. haematobium* y *S. mansoni*para formular pautas concretas sobre el uso de dosis repetidas de 40 o 60 mg/kg para el tratamiento de esquistosomiasis. Aconsejamos el uso de la fórmula de la prueba de reducción del conteo de huevos (RCH) recomendada por la OMS para evaluar la eficacia del praziquantel y que los resultados puedan compararse en diferentes regiones.

Translated from English version into Spanish by Agostina Pan and Maria Paula Gorgone, through

TRANSLATORS
WITHOUT BORDERS
