# Supplementary material for: Efficacy of praziquantel treatment regimens in pre-school and school aged children infected with schistosomiasis in sub-Saharan Africa: a systematic review
Source: Infect Dis Poverty. 2018 Jul 5;7:73. doi: 10.1186/s40249-018-0448-x (PMC6036702; doi:10.1186/s40249-018-0448-x)
Supplement: Supplementary file 2 — Table S1. Summary of findings on the use of praziquantel against Schistosoma mansoni between 2008–2017 in sub-Saharan Africa. (DOCX 25 kb) [file 40249_2018_448_MOESM2_ESM.docx]

Table 1: Summary of review of findings on the use of praziquantel against *Schistosoma mansoni* between 2008-2017 in sub-Saharan Africa

| Author [reference] | Objective(s) | Age group of Study Population/Country of study area | Population size | Species of *Schistosoma* | Type of study | Dosage of praziquantel and time of assessment | Findings |
| --- | --- | --- | --- | --- | --- | --- | --- |
| Sousa-Figueiredo et *al.* 2010 [17] | - To identify the best diagnosis of intestinal schistosomiasis; - To investigate treatment safety and efficacy; - To extend the current WHO dose pole for chemotherapy. | ≤5 years old/Uganda | 363 | *Schistosoma mansoni* | Interventional | 40 mg/kg/3 weeks (21 days) | - 62·3% of the children were confirmed to be infected. - One day after treatment, the following side effects were reported: headaches (3-6%), vomiting (9-4%), diarrhoea (10·9%) and urticaria (8·9%). - Parasitological cure rate was found to be 100·0%   The current dose pole that includes two new height-intervals: 60–84 cm for one-half tablet and 84–99 cm for three-quarter tablet divisions resulted in 97·6% of children receiving an acceptable dose of 30–60 mg/kg. |
| Muheki et *al.* 2011 [18] | To compare the efficacy of two doses versus one dose of  praziquantel on schistosomiasis infection and related morbidity | >6 years/Uganda | 446 | *Schistosoma mansoni* | Interventional | 40 mg/kg single dose and 40 mg/kg repeated at 2 weeks interval /9 weeks after initial dose | - Cure rates of single and double dose treatment groups were 47.9% and 69.7% respectively. - The egg reduction rate of a single dose (92.3%) was not significantly different from two repeated doses (93.9%).   The prevalence of splenomegaly, hepatomegaly and hepatosplenomegaly were 4.9%, 24.2% and 30.3% respectively while periportal fibrosis was minimal. |
| Haile et al. 2012 [19] | To determine prevalence and evaluate the effectiveness of praziquantel against *S. mansoni* | 6-14 years/Ethiopia | 204 | *Schistosoma mansoni* | Interventional | 40 mg/kg of praziquantel/4 weeks post treatment | - Prevalence of S. mansoni was 67.6%. - Cure rate and egg reduction rate were 80.9 and 99.51%, respectively. |
| Navaratnam et *al*. 2012 [20] | To assess the safety and efficacy of praziquantel (PZQ) syrup in comparison with crushed PZQ tablets against intestinal schistosomiasis in preschool children | ≤5 years /Uganda | 1144 | *Schistosoma mansoni* | Interventional | 40 mg/kg/ 3 weeks post-treatment | - Cure rates were 80.9% for the PZQ syrup arm and 81.7% for the crushed PZQ tablet group, with egg reduction rates of 86.1% and 89.0%, respectively. - Pre-treatment infection intensity influenced cure rates significantly. Cure rates of 88.6% in light infections, 74.5% in moderate infections and 67.4% in heavy infections were observed. |
| Sousa-Figueiredo et *al*. 2012 [21] | To assess the efficacy and safety of PZQ treatment in under seven year olds living in *Schistosoma*  *mansoni* endemic areas. | ≤ 7 years /Uganda | 303 | *Schistosoma mansoni* | Interventional | 40 mg/kg /3-4 weeks post-treatment | - Cure rate (56.4%) was found to be significantly different between sub-sets of children who had a history of multiple praziquantel treatments. It was 41.7%, in those that received between one and four in an 18 month period and 77.6% in those that had never received treatment. - Egg reduction rate was 92.1% in those who had received multiple round of praziquantel and 99.1% in those who had not received any treatment. - Praziquantel showed to be safe, with only mild reported side effects. |
| Nalugwa et *al.* 2015 [22] | To compare the impact of single and double dose praziquantel (PZQ) treatment on cure rates (CRs), egg reduction rates (ERRs) and re-infection rates 8 months later among preschool-aged children in Uganda | 1-5 years /Uganda | 1017 | *Schistosoma mansoni* | Randomised control trial | 40 mg/kg single dose and repeated dose /4 weeks post treatment | - CRs were not significantly higher in children treated with two 40 mg/kg PZQ doses (85.5%; 290/339) compared to a single dose (83.2%; 297/357) within 4 weeks. - ERRs were significantly higher with the repeated dose: 99.3% (95%CI: 99.2-99.5) compared to 98.9 (95%CI: 98.7-99.1) using a single dose, (P = 0.01). - Side effects were mild and transient. - Re-infection rate at 8 months after treatment was 44.5%. |
| Crellen et *al.* 2016 [23] | To examine the efficacy of praziquantel among children infected with *Schistosoma mansoni* from 6 schools that had previously received 1-9 rounds of mass drug administration (MDA) with praziquantel in Uganda. | 6–12 years school children /Uganda | 414 | *Schistosoma mansoni* | Interventional | 40 mg/kg /4 weeks post treatment | The average egg reduction rate in schools that had received 8 or 9 previous rounds of mass drug administration (MDA) (88.23%–93.64%) was significantly lower than the average in schools that had previously received 5 rounds (96.13%–99.08%) or 1 round (95.51%–98.96%) of MDA. |
| Coulibaly et *al.* 2017 [9] | To assess the efficacy and safety of escalating praziquantel dosages in preschool-aged children and school-aged children in Southern Ivory Coast | 2–15 years school-aged children /Côte d’Ivoire | 341 | *Schistosoma mansoni* | Randomised control trial | 20-40-60 mg/kg /3 weeks post treatment | 1. In preschool-aged children, cure rates were as follows:  - 62% at 20 mg/kg dose; - 72% at 40 mg/kg dose; - 71% at 60 mg/kg dose.  1. In school-aged children, cure rates were as follows:  - 30% at 20 mg/kg dose; - 69%; at 40 mg/kg dose; - 83% at 60 mg/k dose.   Adverse events (diarrhoea, abdominal pains, and vomiting) were similar with the three different dosages of praziquantel. |

Table 2: Summary of review of findings on the use of praziquantel against *Schistosoma haematobium* between 2008-2017 in sub-Saharan Africa

| Author (reference) | Objective(s) | Age group of Study Population/Country of study area | Population size | Species of *Schistosoma* | Type of study | Dosage of praziquantel and time of assessment | Findings |
| --- | --- | --- | --- | --- | --- | --- | --- |
| Tukahebwa et *al.* 2013 [24] | To assess the effect of one versus two doses of praziquantel on cure rate and reinfection with *Schistosoma mansoni* in high endemic settings along lake Victoria in Uganda | School-aged children /Uganda | 395 | *Schistosoma haematobium* | Interventional | 40 mg/kg single dose or repeated dose with 2 weeks interval /9 weeks after initial dose | - Those that received 2 doses were more likely to be cured (69.7%) than those who received one dose (47.7%). - The geometric mean intensity at 9 months was 12.0 eggs/gram of faeces among those who received 2 doses and 22.1 among those who received one dose. - Re-infection rate at 8 months post treatment was not significant in both groups. It was 61.6% among those who received 2 doses and 68.3% in those that received a single dose. |
| Ojurongbe et *al.* 2014 [25] | To evaluate the efficacy of 2 doses of oral PZQ for treatment among school children in rural communities of Nigeria | 4 - 15 years /Nigeria | 350 | *Schistosoma haematobium* | Interventional | 40 mg/kg at repeated dose / 4 weeks interval post treatment | - At four, eight and twelve weeks post treatment, the egg reduction rates were 57.1%, 77.6% and 100%, respectively. - The egg reduction rate was significantly higher among light infections compared to those to heavy infections. - After the second round of praziquantel, cure rates at 8 weeks and 12 weeks were 85.3% and 100%, respectively. |
| Senghor et *al.* 2015 [26] | To determine the current prevalence of S. haematobium in children at Niakhar; to assess the efficacy of one dose of praziquantel (40 mg/kg) against *S. haematobium* and to monitor reinfection*.* | 5-15 years /Senegal | 329 | *Schistosoma haematobium* | Interventional | 40 mg/kg /5 weeks post treatment | - The cure rates ranged from 89.4 % to 100 % with the egg reduction rates from 77.6 % to 100 %. - The reinfection rate was 12.6 % 2-3 months later and was significantly higher in male children than in female children. - The overall prevalence became significantly lower (13.8 %) than the prevalence at baseline (73.2 %). |
| Munisi et *al.* 2016 [27] | To compare the efficacy of single dose 40mg/kg against repeated dose 40mg/kg praziquantel on parasitological (egg reduction rate and cure rates) and morbidity indicators | 6 -16 years /Tanzania | 431 | *Schistosoma haematobium* | Randomised control trial | 40 mg/kg single dose and 40 mg/kg repeated/ 8 weeks post-treatment | - At 8 weeks, cure rate was higher among those that received repeated dose (93.10%) than those that received single dose (68.68%) (p<0.001). - The egg reduction rate was also higher among those on repeated dose (97.54%) than on a single dose (87.27 (p=0.0062) - An increase on the mean haemoglobin levels at 8 months was observed. |
| Kabuyaya et *al.* 2017 [28] | To assess the efficacy of PZQ and to determine the re-infection rate of *Schistosoma haematobium* infection among school-going children in the Ndumo area, KwaZulu-Natal | 10-15 years /South Africa | 320 | *Schistosoma haematobium* | Interventional  (cohort) | 40 mg/kg at repeated dose /4 weeks interval post treatment | - After the initial dose cure rates were 88.07% and 82.92% for females and males, respectively; egg reduction rates of 80% and 64% for females and males respectively were observed 4 weeks after the initial treatment. - After the second treatment, CR was 100% in females and 50% in males with an egg reduction rate of 100% in females and 70% in males. - At 20 and 28 weeks post treatment, re-infection rates of 8.03% and 8.00% were observed, respectively. |
