# Supplementary material for: Efficacy of praziquantel treatment regimens in pre-school and school aged children infected with schistosomiasis in sub-Saharan Africa: a systematic review
Source: Infect Dis Poverty. 2018 Jul 5;7:73. doi: 10.1186/s40249-018-0448-x (PMC6036702; doi:10.1186/s40249-018-0448-x)
Supplement: Supplementary file 3 — Table S2. Summary of findings on the use of praziquantel against Schistosoma haematobium between 2008–2017 in sub-Saharan Africa. (DOCX 22 kb) [file 40249_2018_448_MOESM3_ESM.docx]

Table 2: Summary of review of findings on the use of praziquantel against *Schistosoma haematobium* between 2008-2017 in sub-Saharan Africa

| Author (reference) | Objective(s) | Age group of Study Population/Country of study area | Population size | Species of *Schistosoma* | Type of study | Dosage of praziquantel and time of assessment | Findings |
| --- | --- | --- | --- | --- | --- | --- | --- |
| Tukahebwa et *al.* 2013 [24] | To assess the effect of one versus two doses of praziquantel on cure rate and reinfection with *Schistosoma mansoni* in high endemic settings along lake Victoria in Uganda | School-aged children /Uganda | 395 | *Schistosoma haematobium* | Interventional | 40 mg/kg single dose or repeated dose with 2 weeks interval /9 weeks after initial dose | - Those that received 2 doses were more likely to be cured (69.7%) than those who received one dose (47.7%). - The geometric mean intensity at 9 months was 12.0 eggs/gram of faeces among those who received 2 doses and 22.1 among those who received one dose. - Re-infection rate at 8 months post treatment was not significant in both groups. It was 61.6% among those who received 2 doses and 68.3% in those that received a single dose. |
| Ojurongbe et *al.* 2014 [25] | To evaluate the efficacy of 2 doses of oral PZQ for treatment among school children in rural communities of Nigeria | 4 - 15 years /Nigeria | 350 | *Schistosoma haematobium* | Interventional | 40 mg/kg at repeated dose / 4 weeks interval post treatment | - At four, eight and twelve weeks post treatment, the egg reduction rates were 57.1%, 77.6% and 100%, respectively. - The egg reduction rate was significantly higher among light infections compared to those to heavy infections. - After the second round of praziquantel, cure rates at 8 weeks and 12 weeks were 85.3% and 100%, respectively. |
| Senghor et *al.* 2015 [26] | To determine the current prevalence of S. haematobium in children at Niakhar; to assess the efficacy of one dose of praziquantel (40 mg/kg) against *S. haematobium* and to monitor reinfection*.* | 5-15 years /Senegal | 329 | *Schistosoma haematobium* | Interventional | 40 mg/kg /5 weeks post treatment | - The cure rates ranged from 89.4 % to 100 % with the egg reduction rates from 77.6 % to 100 %. - The reinfection rate was 12.6 % 2-3 months later and was significantly higher in male children than in female children. - The overall prevalence became significantly lower (13.8 %) than the prevalence at baseline (73.2 %). |
| Munisi et *al.* 2016 [27] | To compare the efficacy of single dose 40mg/kg against repeated dose 40mg/kg praziquantel on parasitological (egg reduction rate and cure rates) and morbidity indicators | 6 -16 years /Tanzania | 431 | *Schistosoma haematobium* | Randomised control trial | 40 mg/kg single dose and 40 mg/kg repeated/ 8 weeks post-treatment | - At 8 weeks, cure rate was higher among those that received repeated dose (93.10%) than those that received single dose (68.68%) (p<0.001). - The egg reduction rate was also higher among those on repeated dose (97.54%) than on a single dose (87.27 (p=0.0062) - An increase on the mean haemoglobin levels at 8 months was observed. |
| Kabuyaya et *al.* 2017 [28] | To assess the efficacy of PZQ and to determine the re-infection rate of *Schistosoma haematobium* infection among school-going children in the Ndumo area, KwaZulu-Natal | 10-15 years /South Africa | 320 | *Schistosoma haematobium* | Interventional  (cohort) | 40 mg/kg at repeated dose /4 weeks interval post treatment | - After the initial dose cure rates were 88.07% and 82.92% for females and males, respectively; egg reduction rates of 80% and 64% for females and males respectively were observed 4 weeks after the initial treatment. - After the second treatment, CR was 100% in females and 50% in males with an egg reduction rate of 100% in females and 70% in males. - At 20 and 28 weeks post treatment, re-infection rates of 8.03% and 8.00% were observed, respectively. |
