# Supplementary material for: Efficacy of praziquantel treatment regimens in pre-school and school aged children infected with schistosomiasis in sub-Saharan Africa: a systematic review
Source: Infect Dis Poverty. 2018 Jul 5;7:73. doi: 10.1186/s40249-018-0448-x (PMC6036702; doi:10.1186/s40249-018-0448-x)
Supplement: Supplementary file 4 — Table S3. Summary of review of findings on the use of praziquantel on co-infection with Schistosoma haematobium and S. mansoni between 2008–2017 in sub-Saharan Africa. (DOCX 22 kb) [file 40249_2018_448_MOESM4_ESM.docx]

Table 3: Summary of review of findings on the use of praziquantel on co-infection with *Schistosoma haematobium* and *S. mansoni* between 2008-2017 in sub-Saharan Africa

| Author (reference) | Objective(s) | Age group of study population and Country of study area | population size | Species of *Schistosoma* | Type of study | Dosage of praziquantel and time of assessment | Findings |
| --- | --- | --- | --- | --- | --- | --- | --- |
| Mutapi et *al.* 2011 [29] | To investigate the safety and efficacy of praziquantel among pre-school children | 1-5 years /Zimbabwe | 104 | *Schistosoma haematobium* and *S. mansoni* | Interventional (cohort) | 40 mg/kg /6 weeks post-treatment | - Egg reduction rate (ERR) of 99% and cure rate (CR) of 92% were reported and were comparable to the efficacy of praziquantel in 6–10 years children where ERR was 96% and CR was 67%. - Of the 104 children, 3.8% reported side effects within 24 hours of taking PZQ treatment such stomach ache, loss of appetite, lethargy and inflammation of the face and body. |
| Coulibaly et *al.* 2012 [30] | To assess the efficacy and safety of crushed praziquantel tablets among preschool-aged children in the Azaguie district. | <6 years /Ivory coast =Côte d’Ivoire | 160 | *Schistosoma mansoni* and *S. haematobium* | Interventional (cross-sectional) | 40 mg/kg /3 weeks after treatment | - Crushed praziquantel showed high efficacy against both species. Cure rate (CR) of 88.6% and egg reduction rate (ERR) of 96.7% for *S. mansoni*; CR of 88.9% and ERR, 98.0%) for *S. haematobium*. - Generally, praziquantel was well tolerated except few severe adverse sides (body and face inflammation) in 4 children. |
| Garba et *al*. 2013 [31] | To assess the efficacy and safety of two closely spaced doses of praziquantel against *Schistosoma haematobium* and *S. mansoni* infection among school-aged children in western Niger | School-aged children /Niger | 877 | *Schistosoma haematobium* and *Schistosoma mansoni* | Interventional (cohort) | 40 mg/kg at repeated dose /3 weeks post treatment | - Cure rates of praziquantel against *S.* *haematobium*-infection at 3 weeks interval ranged between 49.2% and 98.4% with moderate-to-high egg reduction rates (71.4–100%). - Cure rates and egg reduction rates were only moderate among *S. mansoni* infection |
| Garba et *al*. 2013 [32] | To assess the safety and efficacy of praziquantel syrup (Epiquantel^®^) in preschool-aged children in Niger | ≤ 72 months /Niger | 243 | *Schistosoma haematobium* and *Schistosoma mansoni* | Interventional (cohort) | 40 mg/kg / 3 weeks post-treatment | - Praziquantel syrup showed moderate-to-high efficacy against *Schistosoma haematobium* with egg reduction rates of 69.4% and 71.2% at 3 and 6 weeks post-treatment and cure rates of 85.7% and 94.9 respectively. - Lower cure and egg reduction rates were observed against *Schistosoma mansoni* (e.g. cure rate at 6 weeks was only 50.6%). - Adverse events ranged from abdominal pain, bloody diarrhoea and sleepiness although transient. |
| Tchuenté et *al.* 2013 [33] | To determine the performance of praziquantel against mixed infection foci for *Schistosoma* in northern Cameroun | School-aged children /Cameroun | 1000 | *Scistosoma mansoni* and *Schistoma haematobium* | Interventional (cohort) | 40 mg/kg single dose or at repeated dose with 3 weeks interval /6 weeks post treatment | - Cure rates against Schistosoma species in 2 mixed settings were 83.3% (in Bessoum) and 89.0% (in Ouro Doukoudje) while it was 95.3% in a single infection (Makenene). - No case of mixed schistosome infection was recorded after treatment. - Cure rate for *S. mansoni* varied from 99.5% to 100% while it was lower for *S. haematobium* (82.7% -88.0%). - Re-infection rates were higher for *S. haematobium* than for *S. mansoni.* |
| Webster et *al.* 2013 [34] | To investigate the transmission and re-infection dynamics in mixed foci of *S. mansoni* and *S. haematobium* along Senegal basin river | 5-15 years /Senegal | Not specified | *Schistosoma mansoni* and *Schistosoma haematobium* | Interventional (cohort) | 40 mg/kg at repeated dose /3 weeks post treatment | - After the first round of paziquantel sufficient cure rates for both *Schistosoma mansoni* and *S. haematobium* were achieved in all villages (38–96%) with high egg reduction rates (97–99%). - High and rapid re-infection rates occurred, especially for *S. mansoni* six months post treatment. |
